# Influence of Diatomaceous Earth Particle Size on Mechanical Properties of PLA/Diatomaceous Earth Composites

**DOI:** 10.3390/ma15103607

**Published:** 2022-05-18

**Authors:** Marta Dobrosielska, Renata Dobrucka, Dariusz Brząkalski, Miłosz Frydrych, Paulina Kozera, Monika Wieczorek, Marek Jałbrzykowski, Krzysztof J. Kurzydłowski, Robert E. Przekop

**Affiliations:** 1 Faculty of Materials Science and Engineering, Warsaw University of Technology, ul. Wołoska 141, 02-507 Warsaw, Poland; marta.dobrosielska@pw.edu.pl (M.D.); paulina.kozera@pw.edu.pl (P.K.); monika.wieczorek.dokt@pw.edu.pl (M.W.); 2 Department of Non-Food Products Quality and Packaging Development, Institute of Quality Science, Poznań University of Economics and Business, al. Niepodległości 10, 61-875 Poznań, Poland; 3 Faculty of Chemistry, Adam Mickiewicz University in Poznań, ul. Uniwersytetu Poznańskiego 8, 61-614 Poznań, Poland; d.brzakalski@gmail.com (D.B.); frydrych@amu.edu.pl (M.F.); 4 Faculty of Mechanical Engineering, Bialystok University of Technology, ul. Wiejska 45 c, 15-351 Bialystok, Poland; m.jalbrzykowski@pb.edu.pl (M.J.); krzysztof.kurzydlowski@pw.edu.pl (K.J.K.); 5 Centre for Advanced Technologies, Adam Mickiewicz University in Poznań, ul. Uniwersytetu Poznańskiego 10, 61-614 Poznan, Poland

**Keywords:** diatomaceous earth, polylactide, particle size, mechanical properties, biocomposite

## Abstract

The fractionation of diatomaceous earth (DE) using sedimentation made it possible to obtain separate unbroken diatom fractions from broken or agglomerated bodies with a range of particle sizes. The produced filler was used to prepare polylactide (PLA)/diatomaceous earth biocomposite samples containing different particle sizes, which were subjected to mechanical testing (tensile strength, flexural strength, impact strength), colloidal testing (contact angle, color change test, SEM/EDS), and thermal testing (TGA, DSC, DMA). Modification of the PLA containing the smallest particle size with diatomaceous earth (Fraction 5) resulted in a higher impact strength compared to both the pure PLA and the PLA/DE composite that contained base diatomaceous earth. Furthermore, the melt flow rate was improved by more than 80 and 60% for the composite modified with fractionated diatomaceous earth (Fraction 4) compared to pure PLA and base diatomaceous earth, respectively. The elasticity of the composite was also improved from 3.3 GPa for pure polylactide to 4.4 GPa for the system containing the smallest diatomaceous earth particles (Fraction 5).

## 1. Introduction

For many years, an intense increase in the global production of plastic has been observed. This is due, among other factors, to the easy access to their raw materials, improved living standards, and thus a higher consumption. Domka [1] identified that the main objection to the production of plastics is the problem of their resistance to biodegradation and, hence, they remain unchanged in landfills for centuries. In the next few years, the plastics industry is expected to face challenges that are posed by the amendments to relevant directives, such as the requirement to reduce the environmental impact of certain plastic products, especially single-use plastics (SUP). Moreover, societal engagement and pro-environmental action can effectively counteract destruction to the environment [2]. The initiatives presented in the relevant directive are also seen in the wider context of the European Union’s transition toward the idea of a circular economy, which is one of the key areas for the development of the European Union. 

Degradation of the natural environment, development of climate policies, new pro-environmental regulations, and increasing consumer awareness/expectations are changing how business is conducted. A circular economy linked to biological and technical cycles is guided by the following three main principles:

- Preserving and enhancing natural capital by the control of limited resources and the balancing of renewable resource flows;
- Optimizing raw material use by keeping products, components, and materials in circulation with their highest utility in both cycles;
- Developing system efficiency by identifying and removing negative externalities.

The developed concept of the circular economy is, therefore, an approach in which products and materials remain in circulation as long as possible, resulting in reduced waste at landfills. In response, the search for new materials to replace conventional thermoplastics has begun. 

A ubiquitous thermoplastic, with many applications, that requires a reduction in its environmental impact is PLA. The latter is a biodegradable polymer derived from renewable resources with very attractive properties (good mechanical properties, biocompatibility, and good transparency) [3,4]. The main disadvantages of PLA include: rigidity, brittleness, possibility of a partial degradation during processing, the need for drying before processing, and a low resistance to deformation at higher temperatures [5]. To use PLA in certain applications, the properties of such a polymer are modified using various techniques, methods, and technologies. The properties of PLA can be modified by introducing different types of fillers and plasticizers [6], of either natural or synthetic origin. Examples of polylactide reinforced with, for example, natural fibers of various types [7], natural and synthetic zeolites [8], or carbon nanotubes [9] are described in the literature. Examples of synthetic additives to PLA, such as polyadipates, epoxidized oils, citrate esters, poly (ethylene glycol), and lactic acid oligomers, can be found in the literature [10]. Polylactide could also be modified with metallic particles such as silver, magnesium, iron and steel, silicon, boron nitrile, hydroxyapatite, and other alternatives [11,12,13]. In addition, polylactide can be successfully modified with nanoparticles and made into films and used as packaging material in the food industry. For example, inorganic metal/metallic oxide, graphite and silica-based nanoparticles [14], zinc oxide (ZnO) nanofiller [15], nanoclay, nanocelluloses, carbon nanotube, and graphene [16] are used to produce PLA nanocomposites.

In this study, a naturally occurring filler (i.e., diatomaceous earth) is used. With this in mind, our paper acts as a response to the search for suitable alternatives to conventional polymer composites. Diatomaceous earth is chosen because of its unique properties and wide availability. Diatomite is characterized by its porous structure, developed specific surface area, and high silica content [17,18]. Moreover, diatomaceous frustules have a variety of particle sizes, averaging from 3 μm to 200 μm and sometimes up to 1 mm. In raw diatomite, there are both broken, unbroken frustules, and agglomerates particles [19]. Composites with diatomaceous earth are used in many fields, for example, in the packaging industry due to their reinforcement properties, as well as limiting diffusion of gases into the packaging [20]. Diatomaceous earth is also widely used in applications such as a filter material, an additive for dental fillings, a natural insecticide, an additive for membranes [21], and drug carriers [22]. To obtain individual fractions of diatomaceous earth, a sedimentation process is performed, which involves the time-dependent rate of descent of diatoms. Large particles drop more quickly, while smaller particles remain in suspension much longer, which enables their separation. 

In previous papers [23,24], we focused on the creation of epoxy resin/diatomaceous earth composites, which allowed for an observation of the effects of the modifier on changes in the parameters of the uncured epoxy resin. Moreover, we investigated the effects of diatomaceous earth on the process of manufacturing composites and related difficulties. In this paper, we focus on polylactide-based composites that are modified with fractionated diatomaceous earth. The choice of this polymer is dictated by the desire to produce a fully ecological biocomposite, which enables biodegradation under appropriate environmental conditions and, thus, reduces the environmental impact compared to PP/PE-based composites. PLA/diatomaceous earth composites can potentially be used as packaging materials, cutlery, cups, etc. The rationale for this potential application is two-fold: first, its superior biodegradability creates less environmental pollution and, secondly, the properties of the diatomaceous earth naturally imply that the product would be completely safe. 

In this study, diatomaceous earth is fractionated using a sedimentation process and the fractions that result are then used to modify the polylactide and create composites. Composites that contain four different diatomaceous earth particle sizes (fraction 2, fraction 4, fraction 5, and base) and four different modifier concentrations (2.5%, 5%, 10%, and 15%) are obtained. Next, particle size tests (Dynamic Light Scattering—DLS) are conducted and SEM images of the sediments after fractionation are produced. In addition, mechanical, rheological, surface (contact angle), and ageing tests are performed on the composites.

## 2. Materials and Methods

### 2.1. Materials

Polylactide (PLA)-type Ingeo 4043D was purchased from NatureWorks (Minnetonka, Minneapolis, MN, USA). Diatomaceous earth (Perma-Guard, Bountiful, UT, USA) was derived from diatomite deposits. Synthetic wax WTH-B microbeads were bought from WTH GmbH (Hamburg, Germany).

### 2.2. Preparation of Samples

#### 2.2.1. Preparation of Diatomaceous Earth

**Figure 1:**
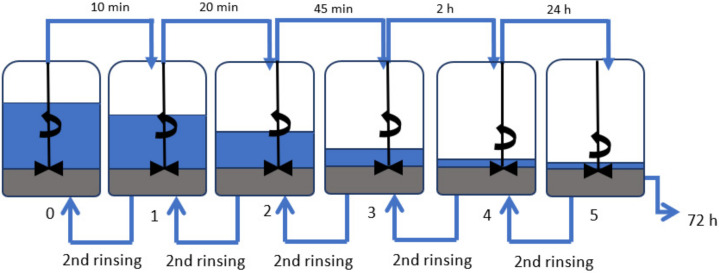
Diagram of diatomaceous earth fractionation.

Amorphous diatomaceous earth (Perma-Guard, Bountiful, UT, USA) (28 kg) was placed into Barrel 3 that can hold 180 L, which was then filled with demineralized water to approximately 100 L. The suspension was then mechanically stirred for 3 min (at a speed of 2000 rpm) and left to undergo sedimentation. After 2 h, the supernatant liquid was pumped into Barrel 4 by using a water pump and again left to undergo sedimentation; Barrel 3 was refilled with demineralized water to about 120 L. After 24 h, the supernatant liquid from Barrel 4 was transferred to Barrel 5. The suspension in Barrel 3 was mechanically stirred and, after 2 h, the supernatant liquid was pumped into Barrel 4. The sediment remaining in Barrel 3 was put into Barrel 2, and Barrel 2 was then filled with demineralized water of volume 140 L. As shown in Figure 1, the process was repeated until each sediment had been washed twice. After this event, the sediments were moved into barrels with lower numbers, while the supernatant liquid was pumped into barrels with higher numbers. At the end of the process, the remaining supernatant liquids were decanted, while the sediments were left to dry at 60 °C for 24 h. The obtained filler fractions together with masses of the sediments are shown in Table 1. The material loss of 1358 kg, with an initial input of 28 kg of diatomaceous earth, arises because the smallest particles of diatomite do not sediment completely and they are removed together with the discarded supernatant liquid when pumping out. 

The following diatomaceous earth fractions were selected for further testing: base, Fraction 2, Fraction 4, and Fraction 5. The detailed study of these fractions is given in Section 3.1. Characterization of the fillers. Base diatoms have every type of frustules (broken, unbroken, and agglomerates) and the proportion of the largest number of particles is average compared to fractionated diatoms. Fraction 2 was chosen because of the absence of agglomerates and an average particle size larger than the others, but also due to the relatively high percentage of sediment mass that is available for further testing. Fractions 4 and 5 have the smallest particle sizes but differ in both small and large particles. This choice of sediments enabled the possibility of testing the properties of the diatomaceous earth, which comprehensively depends on the particle size. 

#### 2.2.2. Preparation of Granulates

The ZAMAK MERCATOR WG 150/280 laboratory mill (Skawina, Poland) was used to homogenize polylactide (PLA 4043D) with base and fractionated diatomaceous earth. For this purpose, 500 g of PLA was plasticized at 210 °C for 15 min with diatomaceous earth added in portions so that the concentrations of 5, 10, 20, and 30% and 1% of WTH-B Microbeads synthetic wax were obtained. Wax-free systems were also prepared for comparison purposes. The systems were then ground using the WANNER C17.26 sv mill (Łódź, Poland) and dried for 24 h at 60 °C. 

#### 2.2.3. Preparation of Final Samples 

The prepared masterbatches were diluted 1:1 with PLA using the Engel e-victory 170/80 (Warsaw, Poland) injection molding machine. Table 2 shows the injection parameters. A holding pressure of linear increment over time was applied. The mold temperature was maintained at 80 °C. Test specimens for the mechanical testing, which accords with EN ISO 20753:2019-01, were obtained. The final concentrations of the systems are shown in Table 3.

### 2.3. Characterization Methods

For flexural and tensile strength tests, the materials obtained were printed into type-1B dumbbell specimens, in accordance with EN ISO 527:2012 and EN ISO 178:2006. The tests of the obtained specimens were performed on the INSTRON 5969 universal testing machine (Instron, Norwood, MA, USA) with a maximum load force of 50 kN. The traverse speed for the tensile strength and the flexural strength measurements were set at 2 mm/min. The Charpy impact test (with no notch) was performed on the Instron Ceast 9050 impact-machine (Instron, Norwood, MA, USA) and accorded with ISO 179-1 (ISO 179-1:2010 Plastics—Determination of Charpy impact properties—Part 1: Non-instrumented impact test, PKN, 2010). The powder morphology was characterized by a scanning electron microscope (SEM, TM 1000 Hitachi, Tokyo, Japan) operating at an applied voltage of 5 kV. Before observations via the SEM, the samples surfaces were sputtered with a gold-palladium layer for 90 s at an electric current of 10 mA and voltage of 2 kV. The size of the diatoms used to prepare the composites was measured with a Mastersizer 3000 (Malvern Instruments Ltd., Malvern, UK). The measurements were taken for samples in water suspension (Hydro EV attachment). The parameters of the measurements for the wet samples were a stirrer revolution speed of 2330 RPM and an ultrasound power of 70%. Contact angle analyses were performed by use of the sessile drop technique (5 μL) at room temperature and atmospheric pressure, with the Krüss DSA100 goniometer (Krüss Optronic GmbH, Hamburg, Germany). Ageing tests were performed in the ESPEC ARS-0220 environmental stress chamber using 10 heating and cooling cycles of −10 °C + 50 °C at an 85% humidity. The melt flow rate (MFR) was measured using the Instron CEAST MF20 melt flow tester (Instron, Norwood, MA, USA), which accords to EN ISO 1133 at 210 °C for a load of 2.16 kg. The thermal properties of the materials were studied using a Q1000 Differential Scanning Calorimeter (TA Instruments, New Castle, DE, USA). Samples with a weight of 8.0 + 0.2 mg were placed into an aluminum hermetic pan. First, the samples were equilibrated at −90 °C, then heated to 280 °C with a scan rate of 10 °C/min, and cooled to −90 °C with a scan rate of 10 °C/min. Finally, they were again heated to 280 °C with a scan rate of 10 °C/min. The process was conducted in a nitrogen atmosphere. Using Universal V4.5A TA software (TA Instruments, New Castle, DE, USA), the glass transition temperature (T_g_) was determined as the midpoint of the glass transition temperature range. The cold crystallization temperature (T_c_) and melting temperature I were taken as the peak temperatures of the cold crystallization and melting, respectively. The measurements for the hiding power were performed by placing samples of the prepared resin systems into the optical path between the light source (LED) and the UV-NIR spectrophotometer, a AvaSpec-Mini2048CL (Avantes, Louisville, CO, USA). Dynamic mechanical analysis (DMA) was performed using a Q800 DMA (TA Instruments, New Castle, DE, USA) using a dual cantilever mode that accords to ASTM D4065-01. From each composite, three rectangular specimens, which were 60 mm long and 10 mm wide, were cut and used in the testing. The analysis was conducted from 0 °C to 130 °C, with a heating rate of 3 °C/min at a frequency of 1 Hz and amplitude of 30 µm. The glass transition temperature (T_g_) was determined from the peak value in the storage modulus, the loss modulus, and the damping factor curves.

## 3. Results and Discussion

### 3.1. Characterization of the Fillers

**Figure 2:**
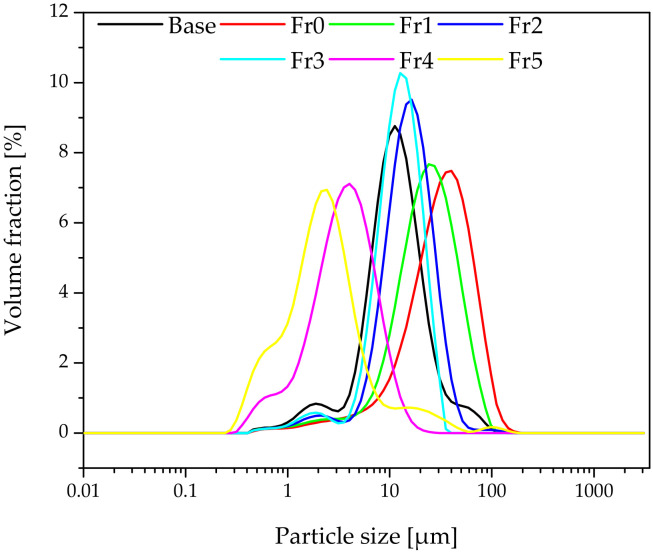
Particle size distribution of individual diatomaceous earth fractions.

**Figure 3:**
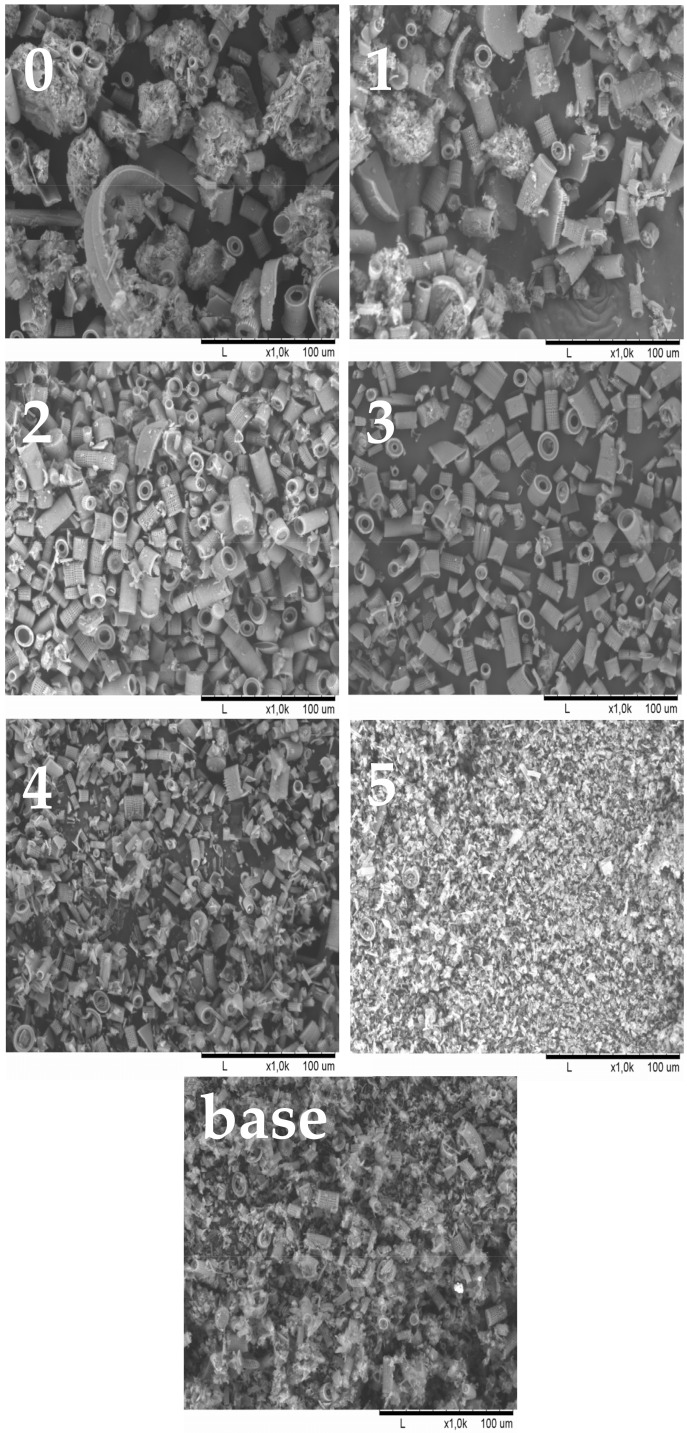
SEM images of fractionated diatomaceous earth; 0—Fraction 0, 1—Fraction 1, 2—Fraction 2, 3—Fraction 3, 4—Fraction 4, 5—Fraction 5, base—base diatomaceous earth.

Figure 2 shows the particle sizes of the fractionated diatomaceous earth. The base diatomaceous earth has broken (0.5–2 µm) and unbroken (2–40 µm) frustules alongside agglomerated particles (above 40 µm). The fractionation process results in both the removal of agglomerated particles and the broken frustules depending on the diatomaceous earth fraction. Furthermore, it can be observed that the longer the sedimentation time, the smaller the diatomaceous earth particles (Fraction 4 and Fraction 5 have sedimentation times of 24 and 72 h, respectively), while short rinsing times enable the extraction of particles with larger sizes (Fraction 0 and Fraction 1 have sedimentation times of 10 and 20 min, respectively). Fractions 2 and 3, which have the respective sedimentation times of 45 min and 2 h, do not differ from the base diatomaceous earth in terms of the small particle size. However, it is possible to eliminate the largest-diameter agglomerates. Some of the agglomerations visible in Fraction 5 arise due to a secondary particle aggregation in the water suspension, which was also observed in the previous report [19]. On the basis of DLS and SEM images (discussed in Section 3.1), the SEM images produced (Figure 3) confirm the relationships mentioned above. As the fraction number increases, the diatomaceous earth particle size decreases and the proportion of the broken frustules increases. The SEM image of Fraction 0 clearly shows that a large number of agglomerates formed for both broken and unbroken frustules. Similarly for Fraction 1, the proportion of the largest particle size is smaller, while Fraction 2 and Fraction 3 have similar particle sizes. It is Fraction 2 that has, by far, the most particles with unbroken frustules, which is consistent with the DLS measurements given above. Fractions 4 and 5, as measured with DLS, show the presence of mostly the smallest particle sizes and a high proportion of broken frustules. For Fraction 5, the presence of such small particles results in the further formation of agglomerates. The base diatomaceous earth contains the combination of all the characteristics described above.

### 3.2. Rheology 

**Figure 4:**
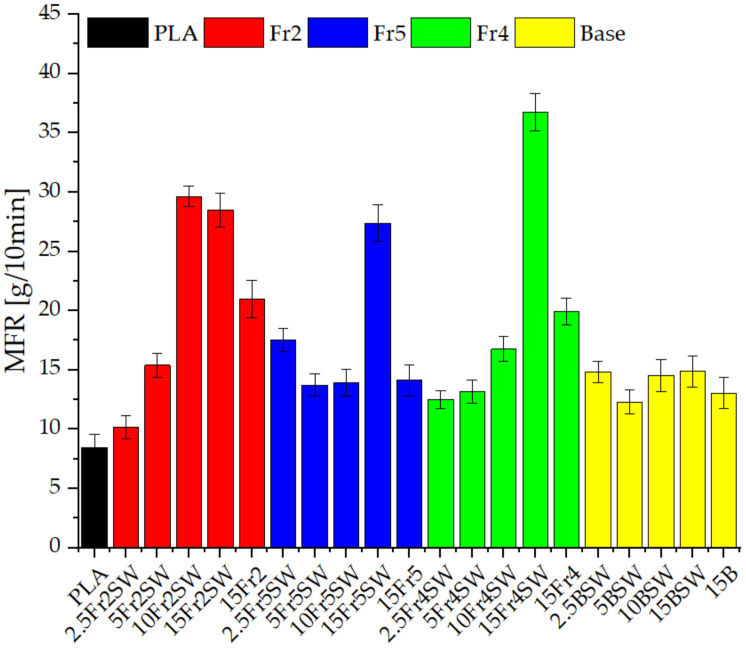
Melt flow rate of granulates.

The melt flow rate was measured for the samples after the injection process (Figure 4). It showed significant differences between the samples that were analyzed. The reference polylactide has an MFR value of approximately 8.5 g/10 min, which is slightly higher than the values found in the literature [25], due to the injection molding. Modifications due to the diatomaceous earth result in a significant increase in the flow rate for all the systems. Fractionated systems with added waxes show a mostly linear increase in the value with an increase in the content of diatomaceous earth. Unmodified diatoms, regardless of their concentration, show similar values at −12–15 g/10 min. For Fraction 2 and Fraction 4, the effect of the addition of the synthetic wax on the MFR values is observed (15Fr2 and 15Fr4 systems compared to 15Fr2SW and 15Fr4SW), i.e., the value increases dramatically (from 20.978, 19.940 to 29.978, 36.747, respectively). Furthermore, the addition of the diatomaceous earth alone increases the melt flow rate, even without the addition of the waxes. This can be seen by comparing the systems that contain 15% diatomaceous earth without wax and with lower concentrations of wax (2.5% and 5%). The diatomaceous earth particle size has a significant effect on the processing parameters. As mentioned above, base diatomaceous earth is characterized by the lack of correlation between its content and an increase or decrease in the MFR, while the system that contains the larger particle sizes (Fraction 2) already clearly display improved properties at a concentration of 10%.

### 3.3. Mechanical Properties

**Figure 5:**
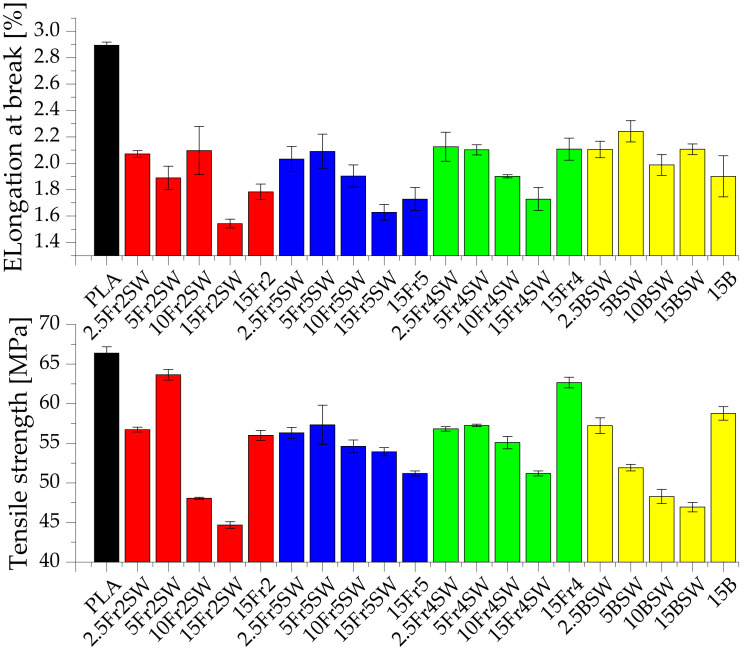
Tensile strength and elongation at break of composites.

**Figure 6:**
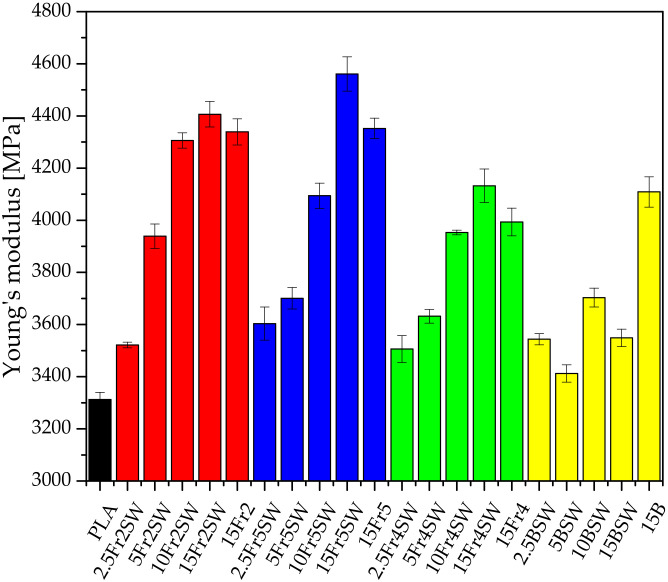
Young’s modulus of composites.

**Figure 7:**
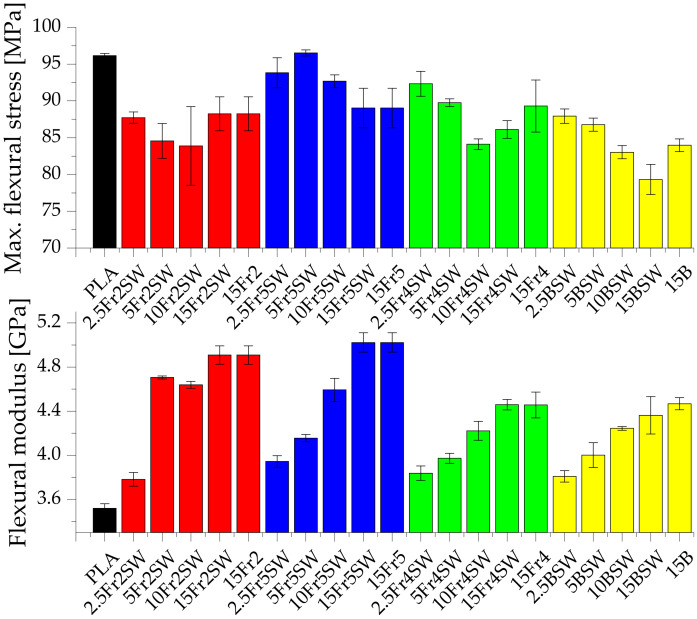
Max. flexural stress and flexural modulus of composites.

A study was conducted to investigate the effect of the particle size on the mechanical properties of the composites (Figure 5, Figure 6, Figure 7 and Figure S1). It is shown that the diatomaceous earth decreases the strength of the material due to, among other factors, the application of rigid particles as the filler, the presence of air in the frustule, and the tendency of the diatomite to agglomerate, which increases the concentration of the point stresses. The last aspect is the lack of chemical surface treatment of the filler, resulting in limited adhesion on the composite interphase. This implies that pure polylactide shows both a higher elongation at break and tensile strength parameters [26]. When using diatomite loadings in the range of −2.5–15%, we can conclude that the optimum amount of additive, for testing mechanical properties at the break point, includes the addition of 5% fractionated diatomaceous earth. However, the base diatomaceous earth shows an inverse relationship between the tensile strength and concentration. The aspects of the PLA-diatoms interaction and the mechanical behavior of PLA-diatomaceous earth, as well as the filler particle size distribution, have been discussed in detail in the previous work [19]. The addition of the synthetic wax (WTH-B) has a significant effect on reducing the mechanical strength of the systems under investigation, while improving the flow rate. The systems modified with Fraction 2 (5% of the additive), i.e., with a medium range of particles but without any agglomerated particles, have the highest parameters, while Fraction 5 (the smallest particles) boasts a relative stability based on their measurements irrespective of the additive concentration.

The flexural strength of the composite systems is also examined. Pure PLA is characterized by the flexural modulus and maximum flexural stress values of 3.52 GPa and 96.15 MPa, respectively. Systems filled with diatomaceous earth, with the addition of synthetic wax, have lower maximum flexural stress values compared to pure polylactide (except for the system 5Fr5SW at 96.53 MPa), which is due to the low adhesion of the polymer to the filler particles. Compared to the tensile tests, the synthetic wax does not cause a significant deterioration in the mechanical parameters relating to flexure. However, the particle size of the filler has a significant effect on the flexural strength of the systems. Fraction 2, which has the highest average particle diameter, causes the greatest decrease in the tensile strength parameters for the highest filling. In each case, the flexural modulus is higher for the filled samples compared to the base PLA (which is typical for filled composites), with an increasing trend with more filling. Moreover, the fractions with the largest and smallest particles show the highest values of the flexural modulus, especially for the highest concentration (15%). The systems that are modified with the base diatomaceous earth, and Fraction 4, show a similar tendency.

**Figure 8:**
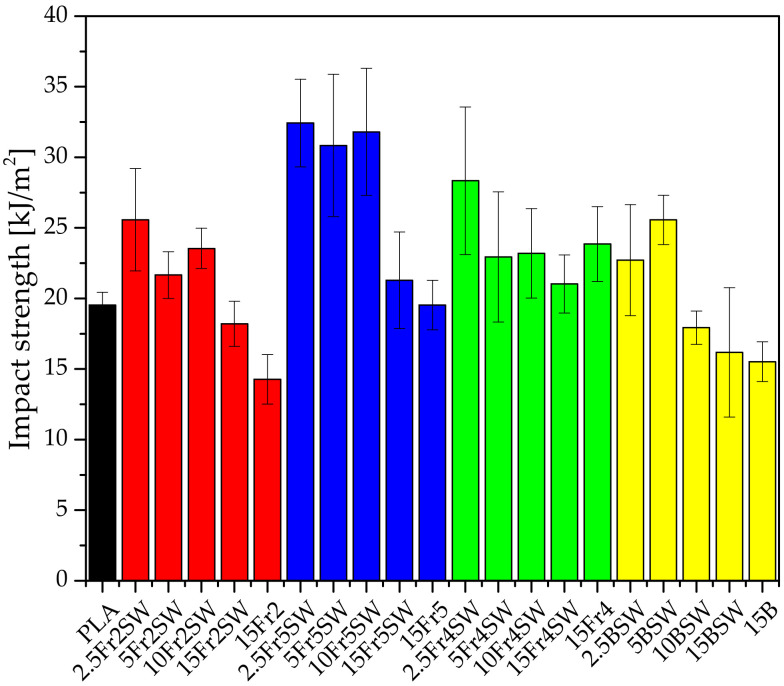
Impact strength of composites.

The addition of diatomaceous earth to the polylactide, irrespective of the fraction, results in an initial increase and then a decrease in the impact strength at most concentrations (Figure 8). The optimum results are obtained for the fraction with the smallest particles, in the filling range up to 10%. The impact strength of the obtained samples is undoubtedly influenced by the particle size of the diatomite and its agglomeration. The fraction that contains the largest-diameter particles has the lowest impact strength parameters, but this is only slightly lower than the systems with the base diatoms, which indicates that the presence of large-diameter particles has a negative effect on the impact strength.

Diatomaceous earth is also shown to affect the brittleness of the material: the higher its addition, the lower the impact strength values. This correlation is the most and least visible for Fraction 5 (the smallest particles) and Fraction 4 (the largest particles, without agglomerates), respectively. In the latter case, the addition of 5% or more does not cause any significant changes, so the impact strength remains at a similar, constant level.

To assess the influence of atmospheric conditions, i.e., the effects of the differences in humidity and temperature on the mechanical and visual properties (Figures S2 and S3), the samples were placed into a climate chamber. Regardless of the fraction used, a decrease in the strength of the composites is minimal for most systems on the addition of wax. In contrast to the systems with wax, the composites without wax show a noticeably higher degradation of their mechanical properties, except for Fraction 5, in which the anti-ageing effect is already caused by the high filler dispersion in the plastic. The correlation between the decrease in the mechanical properties of the samples and the filler concentration (and its fraction) is related to the increased porosity of the samples and, therefore, water has a great impact on them, which result in the hydrolytic degradation of the polylactide under the above-mentioned environmental conditions. The wax acts as a barrier that prevents water from passing through the pores of the filler, as it enables the formation of a hydrophobic layer.

Conditioning the composites in a climatic chamber for 5 days results in a slight increase in the elongation at the break point. However, further exposure of the samples to harsher climatic conditions (for 20 days) cause clear changes in the structure, which manifest in terms of an increased flexibility and, thus, an increased elongation at the break point. The smallest changes are observed, each time, for the highest proportion of the filler, which relates to the lowest light transmission (which is the factor responsible for the photodegradation) within the samples. Photodegradation, together with the hydrolysis, results in a reduction in the average molecular weight of the polymer, which translates into higher sample tensility. The lack of any significant differences between the samples with and without wax is due to a low proportion of photodegradation for samples with a high proportion of the filler. This also indicates that, at higher filling levels, the tensile strength of the samples is so low that their degradation has little to no further effect on this parameter.

Conditioning of the samples in a climate chamber also affects their flexural strength (Figure S4). The flexural modulus, depending on the particle size of the filler used, either remains relatively constant or even increases slightly, as in the case of Fr4 and Fr2 diatomaceous earth systems, or decreases with the time spent in the climatic chamber, as in the case of Fraction 5 and Fraction 2. Compared to pure polylactide, the flexural modulus for the modified samples is maintained at a much higher level, even despite the unfavorable conditioning conditions, indicating the positive effect of the filler on the mechanical properties of the composites, regardless of the particle size. In most cases, the impact strength (Figure S5) reaches its maximum value for samples that are conditioned for 5 days in the climatic chamber, while any further conditioning over the next 20 days partly weakens the structure of the composites, and also lowers the impact strength values, but the results are still higher than for those for unconditioned samples. Young’s modulus decreased slightly for most of the specimens after they were placed in the climate chamber (Figure S6). The optimum results are achieved for systems that contain a 5% addition of diatomaceous earth for the fraction with the smallest particle sizes, i.e., Fraction 5 and Fraction 2, while fractions containing both the larger particle sizes and agglomerates (in the case of the base diatomaceous earth) have significantly lower values. Small-sized particles result in improved energy transfer through the sample. Moreover, the partial degradation of the polymer, which occurs during the first days of the conditioning, causes a reduction in the brittleness of the polymer that, as written above, arises due to the lower average molecular weight. At the highest filling level, in all systems, the presence of the filler reduces the ageing effect on the impact strength of the samples.

### 3.4. Hydrophilic and Hydrophobic Properties of the Obtained Composites

For each system, the maximum contact angle is observed for a given proportion of the filler in the composite. This relates to the balance between the increasing proportion of porous filler and an intensifying micro-roughness of the sample, and the impact of the polar character of the filler surface on the polarity of the composite surface. However, the polar effect is observed to a lesser extent on the presence of wax in the composites. As the size of particle diatoms in the systems increases, the hydrophobicity also increases. The lowest values are observed for the systems that are modified with Fraction 5 (69.3° on average), while for Fraction 4 and Fraction 5, the results are comparable: 86.7 and 84.8° (Table 4). Systems that contain the full range of sizes of particle diatoms, i.e., the base systems, have by far the highest values for the contact angle, which averages to 92.3°. The hydrophobicity of systems, therefore, is affected by both large- and small-sized particles, but a predominance arises for particles above 3 nm. However, an adverse effect on the hydrophilic and hydrophobic properties of the composite surface occurs on addition of synthetic wax. By considering systems with a 15% diatomaceous earth content, the higher contact angle values are obtained for systems that are not modified with synthetic wax (except for the base system, in which the values are slightly higher for the system with wax). 

The exposure of the composites to increased humidity, and variable temperature conditions (within the climatic chamber), causes changes in their properties. The system with the smallest diatomaceous earth particles has a higher contact angle for the whole series, regardless of the modifier concentration. Similarly, for systems that are not modified with synthetic wax, the 15% diatom sample for Fraction 2 achieves the highest contact angle result (of all the tested samples) of 110.05°. Holding the systems in the climate chamber for a longer period of time results in decreased values for the contact angle for most systems, which can be explained by the degradation of the polylactide; the latter is evidenced by the decrease in the contact angle for pure PLA after 20 days from 83.4° to 70.6°. The initial increase in the contact angle is related to the development of a microporous structure on the polymer surface [27]. Further ageing of the polymer leads to the formation of larger breaks and the creation of a greater number of polar groups on the PLA surface.

### 3.5. Electron Microscopy—The Effects of Modifier Particle Sizes on Dispersion within the Polymer Matrix

**Figure 9:**
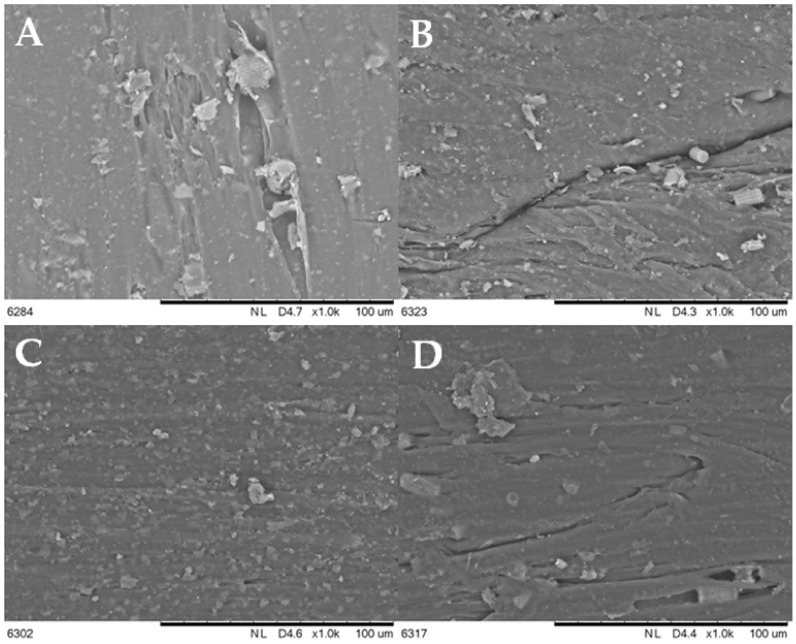
SEM images of various surfaces (**A**–**D**): (**A**)—15Fr2SW, (**B**)—15Fr4SW, (**C**)—15Fr5SW, and (**D**)—15BSW.

Figure 9 shows the images of the composite surface, at magnification of 1000× that derive from the scanning electron microscope (SEM). As mentioned above, Fraction 2 is characterized by the largest diatom particle size, as confirmed by the SEM Figure 9A image. Here, cylindrical diatoms that are surrounded by the polylactide are clearly visible. Similarly for Fraction 4 (Figure 9B), where cylindrical diatoms are also seen, it is clear that they have a slightly smaller size compared to those in Fraction 2. Figure 9C shows Fraction 5, which contains the smallest particle size; here, it can clearly be observed that the diatomaceous earth disperses uniformly within the polymer matrix, and only one secondary agglomerate formation of the diatomaceous earth particles is present. Base diatomaceous earth (Figure 9D), which tends to agglomerate, is not uniformly dispersed within the polymer. 

**Figure 10:**
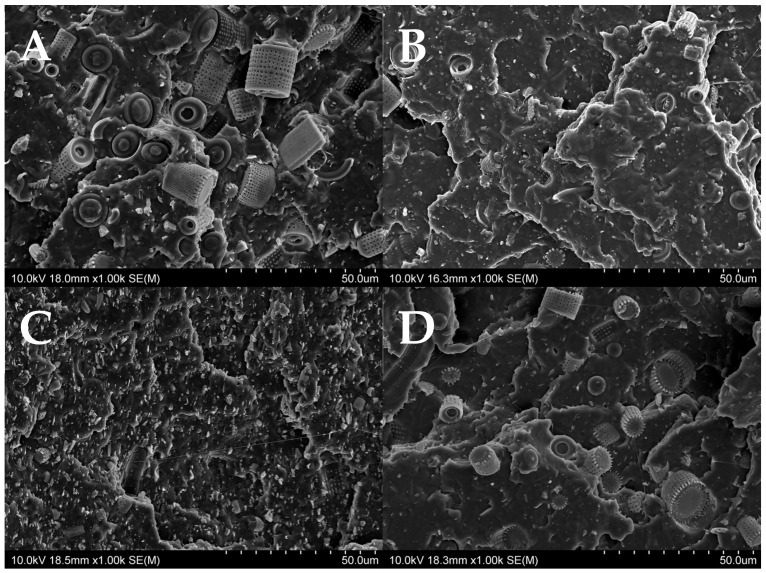
SEM images of the (**A**–**D**) fractures: (**A**)—15Fr2SW, (**B**)—15Fr4SW, and (**C**)—15Fr5SW, (**D**)—15BSW.

The electron microscope is also used to provide images of the fractures that are formed after the impact testing of the composites (Figure 10 and Figure S7). The composite that is modified with Fraction 2 (with the largest particles, see Figure 10A) is characterized by the presence of diatoms with a maximum length of 13 μm and a diameter around 10 μm. Here, whole diatoms are visible, without any broken frustules. The breaking of the test sample does not cause the diatoms to disintegrate into fragments, and no voids remain after diatoms become visible in the matrix. This suggests that the polymer fracture occurs at the filler-polymer interface during a composite failure, rather than a filler pull-out, which is probably due to the low length-to-diameter ratio of the diatom frustules. A 15% concentration for the modifier results in a high local accumulation of the filler, but the agglomeration phenomenon is not observed in this case. The composite that is modified with Fraction 4 (Figure 10B) has a significantly smaller filler size (i.e., a maximum diameter of 9 μm). The proportion of broken diatom frustules is significantly higher than those in Fraction 2, and the imprints in the polymer matrix that result from the pull-out are also present. The composite is modified with diatomaceous earth that has the smallest particle size (Fraction 5, Figure 10C), which are characterized by the presence of the mainly broken diatom fragments; only a small number of whole diatoms are seen and their sizes do not exceed 6 μm. The base diatomaceous earth (Figure 10D) contains both the larger and smaller diatoms.

### 3.6. Hiding Power Tests

**Figure 11:**
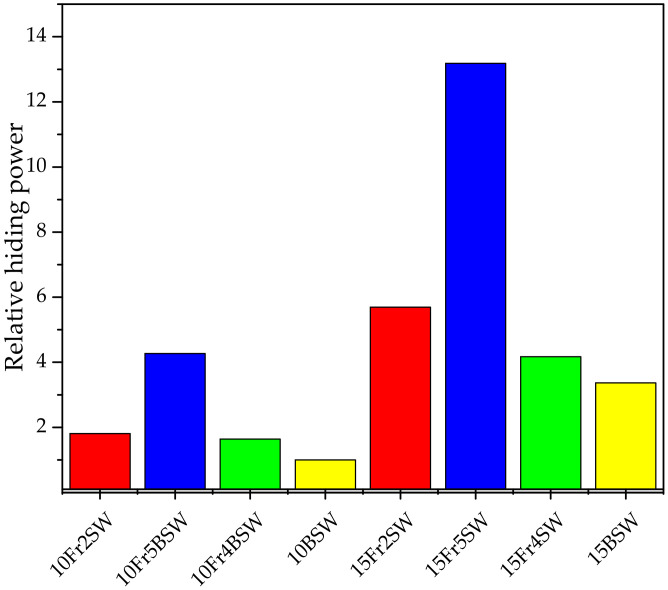
Relative hiding power of the PLA/DE composites.

Hiding power tests, based on the visible light transmittance of the samples, show the impact of both the diatomaceous earth content and the particle size of the individual fractions on the pigmentation efficiency of the composites. The higher the DE content, irrespective of the modifier type, the higher the hiding power. Once again, the highest results are obtained for fractions that contain the diatomaceous earth with the smallest particle size, i.e., Fraction 5. This occurs due to the excellent dispersion characteristics of the particles with small sizes in the polymer matrix, as confirmed by the SEM images (Figure 11). The composites that contain the base diatoms have the lowest hiding power, which is due to the proportion of the agglomerated fraction. A relatively low hiding power is observed for the composites that contain Fraction 4, despite the high proportion of small-diameter particles in the composition.

### 3.7. DMTA Tests

**Figure 12:**
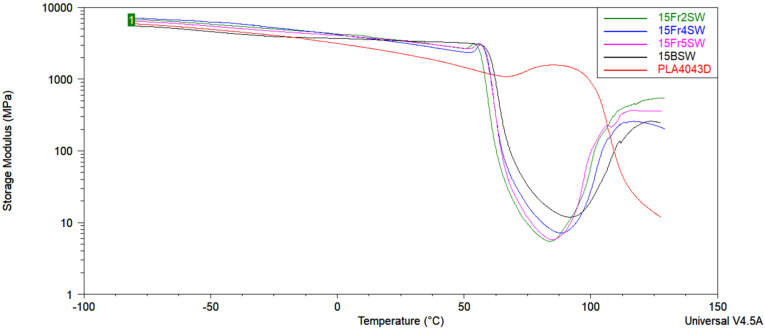
Storage modulus curves of the samples on a logarithmic scale.

Table 5 shows the influences on the storage modulus (E’) due to addition of different volume fractions of diatomaceous earth (Figure 12); this involves the loss modulus (E”), and the damping factor (tan d) for the composites that are based on PLA and diatoms. The T_g_ of neat PLA is less pronounced and observed in the 60–63 °C range. The application of the fractionated diatomite leads to a slight change in the dynamic mechanical properties of the composites. Most importantly, the T_g_ transition is significantly more distinctive. Moreover, it can be observed that the addition of a different fraction of DE enables an increase in the glass transition temperature (T_g_), which is determined from the storage modulus (E’), loss modulus (E”), and the damping factor (tanδ). A sharp decrease in the storage modulus, the loss modulus (E”), and the damping factor (tanδ) of the composites with diatomaceous earth is related to the glass transition of PLA. The glass transition temperature (T_g_) increases slightly when the filler particle size decreases. This shows that the presence of a different volume fraction of DE restricts the segmental motion of the polymer chains.

Based on the DMTA curves at normal scale, cold crystallization and subsequent melting are observed after the glass transition temperature. In the process of cold crystallization, a new fraction of crystallites is formed due to improved mobility and ordering ability of the polymer chains in the amorphous phase, which consequently results in an increase in modulus, and a significant peak in the tan delta curve is observed, with an onset at ~90 °C for PLA composites. This phenomenon was discussed in the review by Cristea et al. [28]. As the DSC and DMTA measurements are based on different measuring principles, the cold crystallization event during the DSC measurement occurs at a slightly different temperature. Crystallites that form at relatively low temperatures during cold crystallization are not stable and undergo crystal perfection. Then, as the temperature increases, the decrease in the modulus indicates the beginning of the crystallite melting process.

### 3.8. Thermal Studies

**Figure 13:**
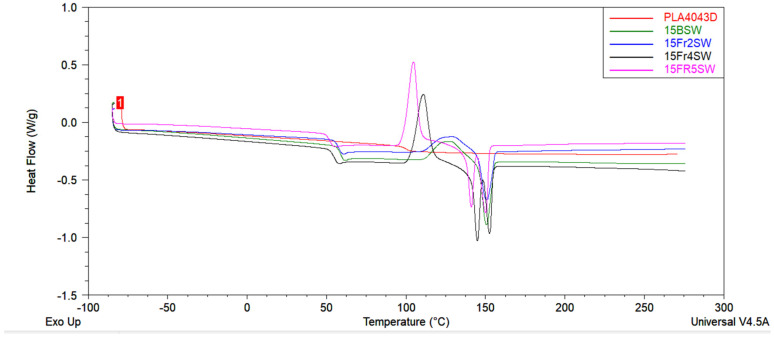
DSC curves of unmodified PLA, and the composites based on PLA, with a different weight fraction of the diatoms obtained during the second heating procedure.

A DSC analysis is performed to investigate the thermal behavior of the PLA and PLA/diatoms composites. Figure 13 shows the DSC thermograms of the PLA and PLA/diatom composites during the second heating stage. When the temperature increases from 52 °C to 153 °C, each DSC curve possesses three thermal characteristics: (a) a glass transition temperature (T_g_) near 55 °C, (b) a cold crystallization peak (T_cc_), and (c) an endothermic melting peak (T_m_).

The values of the glass transition temperature, cold crystallization temperature, specific cold crystallization enthalpy, melting temperature, and the specific melting enthalpy are listed in Table 6. It can be observed that the unmodified PLA and the 15Fr2SW composite have a single melting peak, while the 15Fr4SW and 15Fr5SW composites involve two distinct melting peaks (Tm_1_ and Tm_2_). For the 15BSW and 15Fr2SW materials, there is only one melting peak, and hence, its Tm_1_ equals Tm_2_. Figure 2 and Table 1 reveal that the glass transition temperature decreases with an increase in the weight fraction of the diatoms from 59.3 to 52.1 °C, which indicates that the presence of the diatoms has an effect on the chain mobility of the PLA, by creating more mobile polymer phases on the matrix-filler interphase. This effect is known for composites with no chemical attraction between the polymer matrix and filler surface [29,30,31].

With regard to cold crystallization, Figure 13 shows that the unmodified PLA and the 15Fr2SW composite show a wide cold crystallization peak, which represents its slow crystallization behavior over time; the presence of any diatoms significantly narrows this cold crystallization peak. Moreover, the temperature of the cold crystallization decreases with an increase in the diatom content. Based on these results, it can be determined that the diatoms can greatly promote crystallization of the PLA due to its outstanding nucleating effect.

With a further increase in the temperature, the crystals that are generated during cold crystallization begin to melt. As a result, a melting peak appears in the DSC thermograms. It can be observed that the unmodified PLA and the 15Fr2SW composite show a single melting peak, and its melting temperature (T_m_) is around 150 °C. While the PLA/GF composites reveal a double melting peak with two melting temperatures (Tm_1_ and Tm_2_), the double melting peak could be related to the recrystallization of the generated crystals. For the 15Fr4SW and 15Fr5SW composites that crystallize around 110 °C, the crystals typically show a less-than-perfect structure and they are much thinner than the crystals generated at the higher temperatures during cold crystallization. However, these thinner crystals can melt and recrystallize to generate a closer to perfect and thicker structure. Due to a temperature increase, melting again occurs and the second melting peak (Tm_2_) is achieved [32,33].

## 4. Conclusions

Fractionation of the diatomaceous earth, using the phenomenon of sedimentation, allows for fractions of different grain sizes to become distinguishable. This process enables the separation of broken, unbroken, and agglomerated diatoms. The diatomaceous earth particle size, which is used as a polylactide filler, affects both the rheological and the mechanical properties of the composites. As the filler concentration increases, the melt flow rate (MFR) increases, which varies depending on the diatomaceous earth fraction. It was also determined that the MFR of the unfractionated diatoms has significantly lower values compared to the others. When measuring the tensile strength of the composites, the optimum amount of additive is 5% diatomaceous earth, which produces the highest obtained results; this is regardless of particle size. The highest impact strength is obtained for the composites that are modified with diatomaceous earth that contain the smallest particle size (Fraction 5). The highest hiding degree is achieved for composites that have diatomaceous earth with the smallest particles (Fraction 5). With an increase in the modifier concentration within the system, the hiding degree also increases. The thermal analysis revealed that the particle size of the diatomaceous earth, and its tendency to form agglomerates, both have an effect on the blocking the PLA chain. The addition of synthetic wax provides the PLA composites with diatoms and the resulting anti-ageing properties, which has a positive effect on the mechanical properties after an acceleration of the ageing.

## Figures and Tables

**Table 1:** Percentage of individual diatomaceous earth fractions after fractionation using sedimentation.

	Sediment Mass [kg]	Percentage [%]
Sediment 0	0.592	2.21
Sediment 1	0.206	0.76
Sediment 2	1.934	7.25
Sediment 3	1.778	6.73
Sediment 4	16.730	62.78
Sediment 5	5.402	20.27
In total	26.642	100

**Table 2:** Injection parameters.

**Temperature [°C]**	**Zone 1**	**Zone 2**	**Zone 3**	**Die**
	195.0	200.0	185.0	190.0
**Holding pressure**		**t [s]**	0.0	9.0
		**P [bar]**	500.0	1500.0
**Clamping force [kN]**		**Holding time [s]**	**cooling time [s]**	**screw diameter [mm]**
800		9.0	50.0	25.0

**Table 3:** Final concentrations of the tested systems.

Sample Name	Short Name	Sample Name	Short Name
PLA + 2.5% Fraction 2 + 0.5% WTH-B	2.5Fr2SW	PLA + 2.5% Fraction 4 + 0.5% WTH-B	2.5Fr4SW
PLA + 5% Fraction 2 + 0.5% WTH-B	5Fr2SW	PLA + 5% Fraction 4 + 0.5% WTH-B	5Fr4SW
PLA + 10% Fraction 2 + 0.5% WTH-B	10Fr2SW	PLA + 10% Fraction 4 + 0.5% WTH-B	10Fr4SW
PLA + 15% Fraction 2 + 0.5% WTH-B	15Fr2SW	PLA + 15% Fraction 4 + 0.5% WTH-B	15Fr4SW
PLA + 15% Fraction 2	15Fr2	PLA + 15% Fraction 4	15Fr4
PLA + 2.5% Fraction 5 + 0.5% WTH-B	2.5Fr5SW	PLA + 2.5% base + 0.5% WTH-B	2.5BSW
PLA + 5% Fraction 5 + 0.5% WTH-B	5Fr5SW	PLA + 5% base + 0.5% WTH-B	5BSW
PLA + 10% Fraction 5 + 0.5% WTH-B	10Fr5SW	PLA + 10% base + 0.5% WTH-B	10BSW
PLA + 15% Fraction 5 + 0.5% WTH-B	15Fr5SW	PLA + 15% base + 0.5% WTH-B	15BSW
PLA + 15% Fraction 5	15Fr5	PLA + 2.5% base	15B
PLA			

**Table 4:** Contact angle for the reference samples after 5 and 20 days in the climate chamber (CCh).

	Contact Angle RT [°C]	Contact Angle after 5 Days in CCh [°C]	Contact Angle after 20 Days in CCh [°C]
PLA	83.4	90.5	70.6
2.5Fr2SW	80.4	81.7	82.1
5Fr2SW	84.5	95.6	90.1
10Fr2SW	90.9	87.2	88.6
15Fr2SW	82.8	69.0	89.7
15Fr2	85.7	110.0	74.6
2.5Fr5SW	43.2	76.1	91.0
5Fr5SW	91.5	93.3	89.2
10Fr5SW	80.6	90.8	82.0
15Fr5SW	61.8	72.0	84.0
15Fr5	93.4	95.0	87.6
2.5Fr4SW	86.9	86.5	94.7
5Fr4SW	90.0	83.3	91.8
10Fr4SW	84.7	90.0	80.1
15Fr4SW	85.5	79.5	81.1
15Fr4	92.3	87.3	88.9
2.5BSW	95.6	96.9	77.0
5BSW	94.4	91.2	84.2
10BSW	85.8	38.2	93.5
15BSW	93.3	91.8	96.4
15B	85.7	90.5	87.5

**Table 5:** Summary of the determined values for the glass transition temperature from the DMTA curves.

Sample Name	T_g_ [°C]		
	E’ [MPa]	E” [MPa]	tanδ
15BSW	59.8	61.7	65.2
15Fr2SW	56.7	57.6	62.2
15Fr4SW	58.8	59.8	63.9
15Fr5SW	58.8	59.6	64.4

**Table 6:** Thermal characteristics of the PLA, and composites based on PLA, with a different weight fraction of the diatoms.

Sample	T_g_ (°C)	T_cc_ (°C)	ΔH_cc_ (J/g)	Tm_1_ (°C)	Tm_2_ (°C)	ΔHm (J/g)
PLA	100.0	-	-	-	-	-
15BSW	59.3	127.7	19.0	150.5	150.5	20.4
15Fr2SW	58.2	129.0	16.9	150.6	150.6	17.1
15Fr4SW	55.1	110.8	33.8	144.9	152.6	34.4
15Fr5SW	52.1	104.5	35.0	141.1	150.1	31.5

## Data Availability

Not applicable.

## Supplementary Materials

The following materials are available online at https://www.mdpi.com/article/10.3390/ma15103607/s1, Figure S1: Example of tensile stress–strain curves of composites before climate chamber. Figure S2: Tensile strength for samples before and after 5 and 20 days in climate chamber. Figure S3: Elongation at break for samples before and after 5 and 20 days in climate chamber. Figure S4: Flexural modulus for samples before and after 5 and 20 days in climate chamber. Figure S5: Impact strength for samples before and after 5 and 20 days in climate chamber. Figure S6: Young’s modulus for samples before and after 5 and 20 days in climate chamber. Figure S7: Photograph of fractured specimens after impact test.
