# Supplementary material for: Influence of Diatomaceous Earth Particle Size on Mechanical Properties of PLA/Diatomaceous Earth Composites
Source: Materials (Basel). 2022 May 18;15(10):3607. doi: 10.3390/ma15103607 (PMC9145730; doi:10.3390/ma15103607)
Supplement: Supplementary file 1 [file materials-15-03607-s001.zip › materials-1678394-supplementary.pdf]

materials

MDPI

Supplementary materials

Influence of Diatomaceous Earth Particle Size on Mechanical Properties of PLA/Diatomaceous Earth Composites

Marta Dobrosielska [1], Renata Dobrucka [1,2,*], Dariusz Brząkalski [3], Miłosz Frydrych [3], Paulina Kozera [1], Monika Wieczorek [1], Marek Jałbrzykowski [4,5], Krzysztof J. Kurzydłowski [4] and Robert E. Przekop [5,*]

1 Faculty of Materials Science and Engineering, Warsaw University of Technology, ul. Wołoska 141, 02-507 Warsaw, Poland; marta.dobrosielska@pw.edu.pl (M.D.); paulina.kozera@pw.edu.pl (P.K.); monika.wieczorek@pw.edu.pl (M.W.)
2 Department of Non-Food Products Quality and Packaging Development, Institute of Quality Science, Poznań University of Economics and Business, al. Niepodległości 10, 61-875 Poznań, Poland
3 Faculty of Chemistry, Adam Mickiewicz University in Poznań, ul. Uniwersytetu Poznańskiego 8, 61–614 Poznań, Poland; d.brzakalski@gmail.com (D.B.); frydrych@amu.edu.pl (M.F.)
4 Faculty of Mechanical Engineering, Bialystok University of Technology, ul. Wiejska 45 c, 15-351 Bialystok, Poland; m.jalbrzykowski@pb.edu.pl (M.J.);krzysztof.kurzydlowski@pw.edu.pl (K.J.K.)
5 Centre for Advanced Technologies, Adam Mickiewicz University in Poznań, ul. Uniwersytetu Poznańskiego 10, 61-614 Poznan, Poland
* Correspondence: renata.dobrucka@pw.edu.pl (R.D.) or renata.dobrucka@ue.poznan.pl (R.D.); rprzekop@amu.edu.pl (R.E.P.) or r.przekop@gmail.com (R.E.P.)

Citation: Dobrosielska, M.; Dobrucka, R.; Brząkalski, D.; Frydrych, M.; Kozera, P.; Wieczorek, M.; Jałbrzykowski, M.; Kurzydłowski, K.J.; Przekop, R.E. Influence of Diatomaceous Earth Particle Size on Mechanical Properties of PLA/Diatomaceous Earth Composites. *Materials* **2022**, *15*, 3607. https://doi.org/10.3390/ma15103607

Academic Editor: Sandra Maria Fernandes Carvalho

Received: 28 March 2022
Accepted: 9 May 2022
Published: 18 May 2022

Publisher's Note: MDPI stays neutral with regard to jurisdictional claims in published maps and institutional affiliations.

Copyright: © 2022 by the authors. Licensee MDPI, Basel, Switzerland. This article is an open access article distributed under the terms and conditions of the Creative Commons Attribution (CC BY) license (https://creativecommons.org/licenses/by/4.0/).

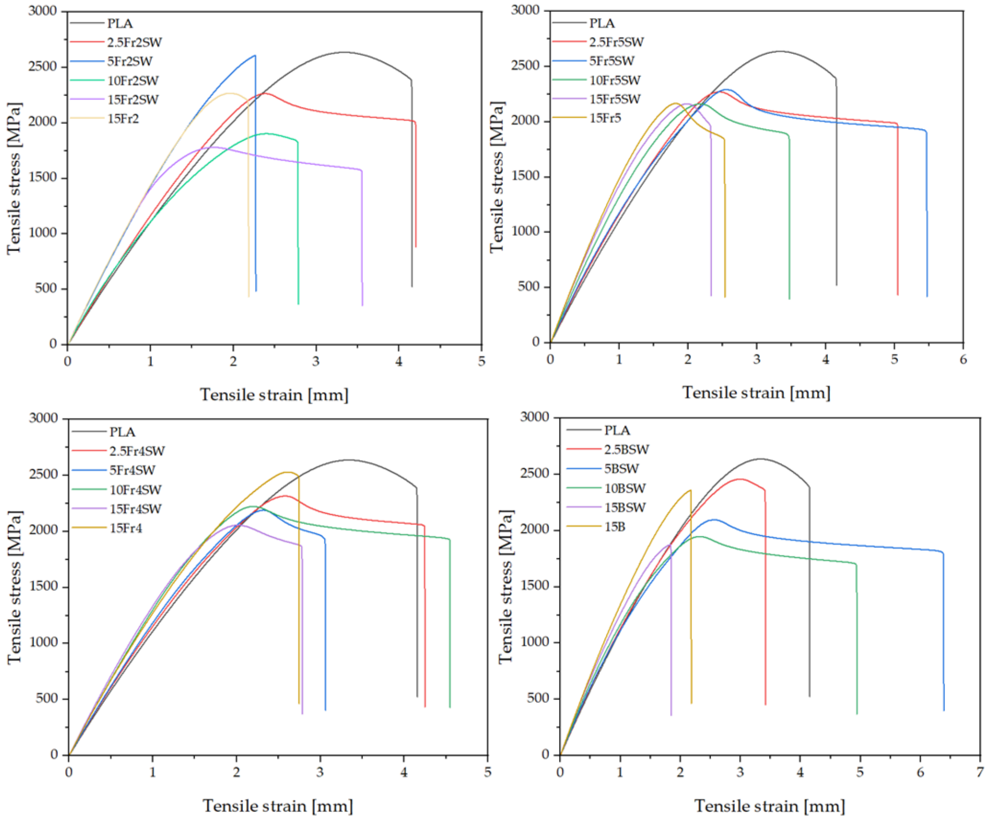

Figure S1. Example of tensile stress–strain curves of composites before climate chamber.

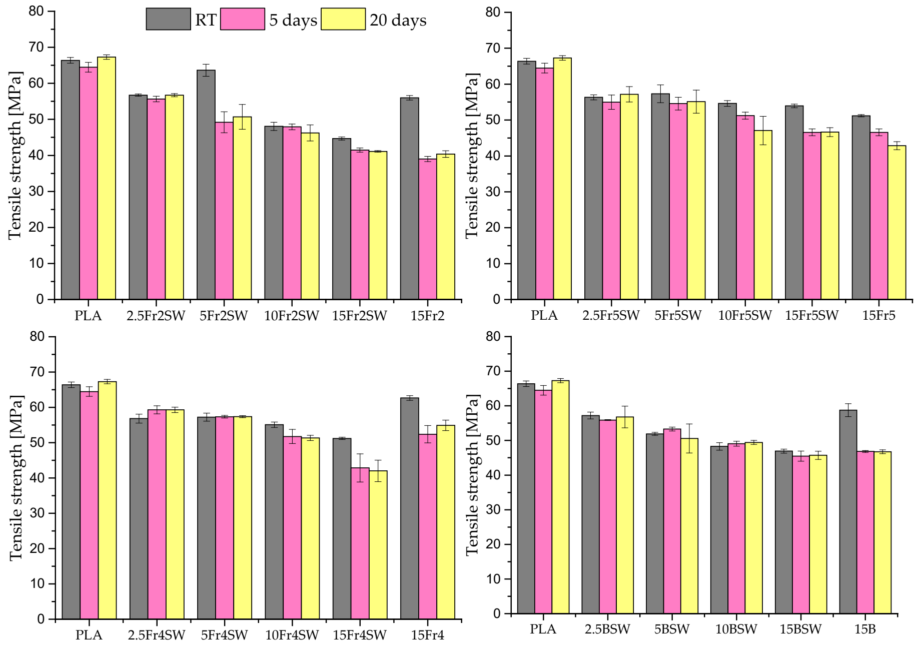

Figure S2. Tensile strength for samples before and after 5 and 20 days in climate chamber.

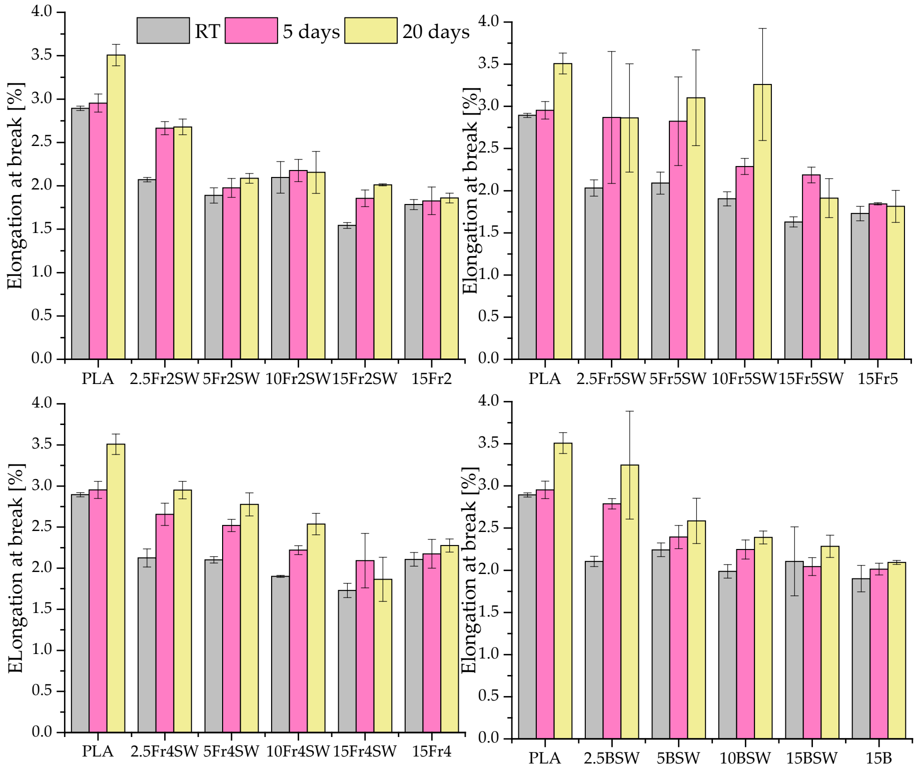

Figure S3. Elongation at break for samples before and after 5 and 20 days in climate chamber.

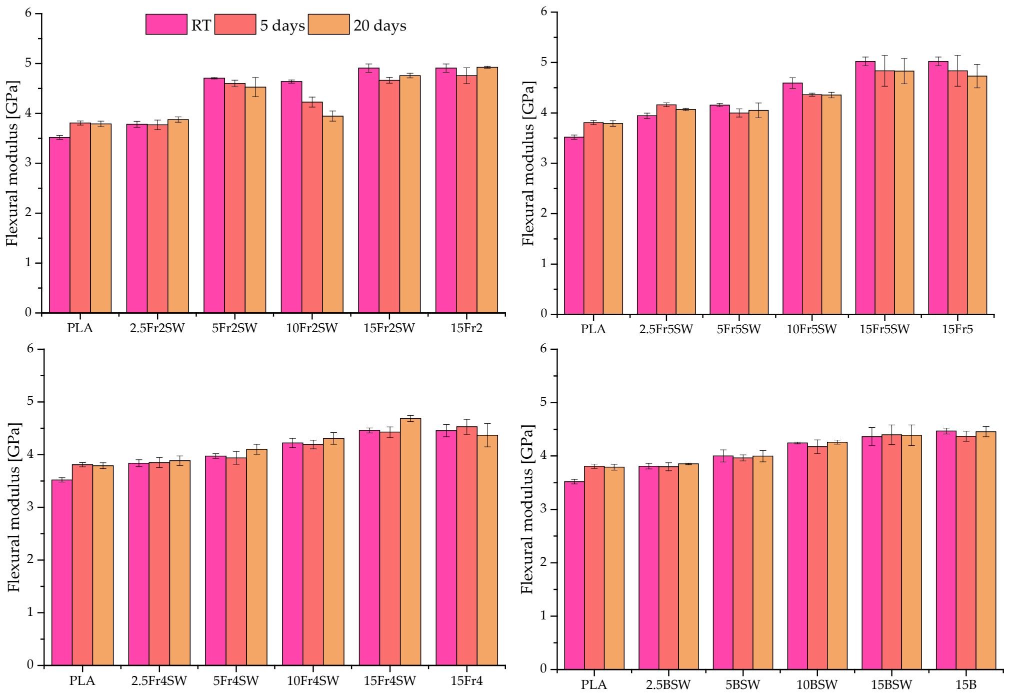

Figure S4. Flexural modulus for samples before and after 5 and 20 days in climate chamber.

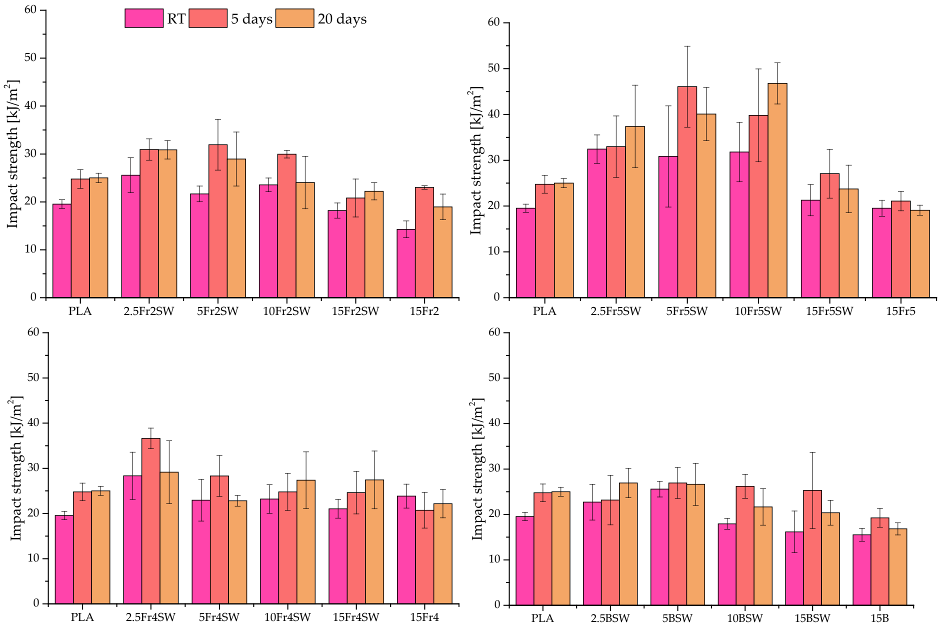

Figure S5. Impact strength for samples before and after 5 and 20 days in climate chamber.

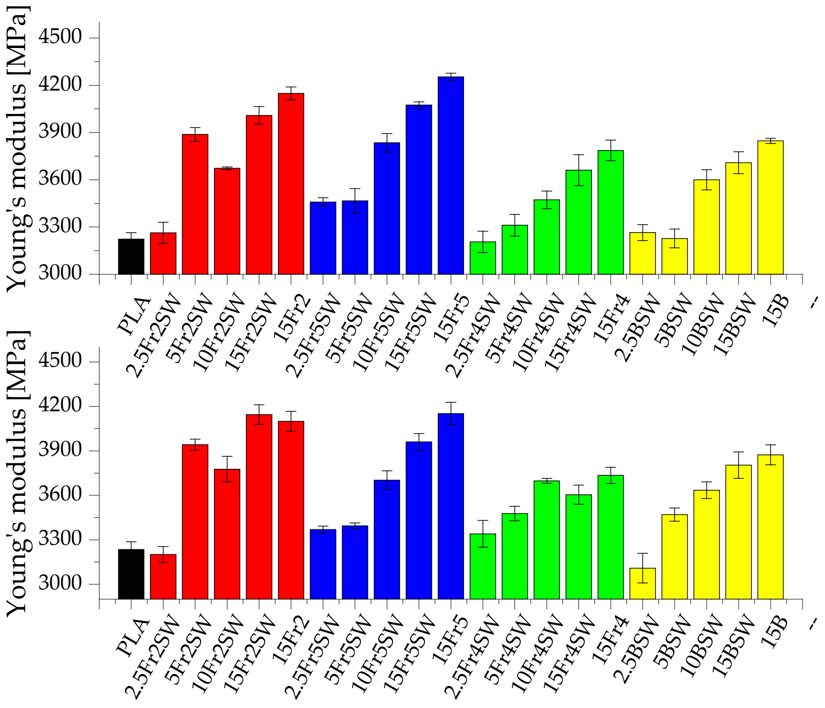

Figure S6. Young's modulus for samples before and after 5 and 20 days in climate chamber.

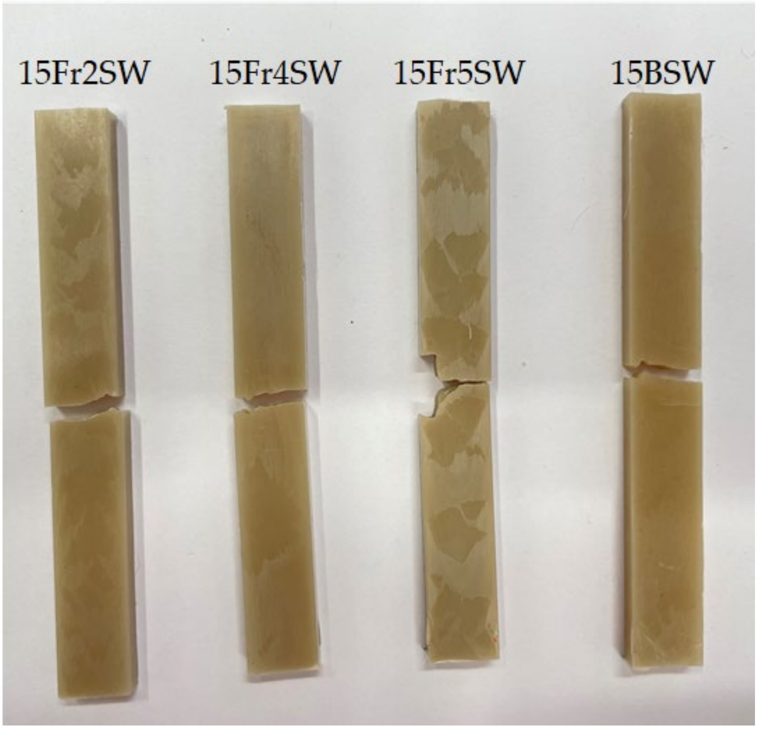

Figure S7. Photograph of fractured specimens after impact test.
